# Genome-Wide Mapping of Yeast RNA Polymerase II Termination

**DOI:** 10.1371/journal.pgen.1004632

**Published:** 2014-10-09

**Authors:** Paul Schaughency, Jonathan Merran, Jeffry L. Corden

**Affiliations:** Department of Molecular Biology and Genetics, Johns Hopkins Medical School, Baltimore, Maryland, United States of America; University of Oxford, United Kingdom

## Abstract

Yeast RNA polymerase II (Pol II) terminates transcription of coding transcripts through the polyadenylation (pA) pathway and non-coding transcripts through the non-polyadenylation (non-pA) pathway. We have used PAR-CLIP to map the position of Pol II genome-wide in living yeast cells after depletion of components of either the pA or non-pA termination complexes. We show here that Ysh1, responsible for cleavage at the pA site, is required for efficient removal of Pol II from the template. Depletion of Ysh1 from the nucleus does not, however, lead to readthrough transcription. In contrast, depletion of the termination factor Nrd1 leads to widespread runaway elongation of non-pA transcripts. Depletion of Sen1 also leads to readthrough at non-pA terminators, but in contrast to Nrd1, this readthrough is less processive, or more susceptible to pausing. The data presented here provide delineation of *in vivo* Pol II termination regions and highlight differences in the sequences that signal termination of different classes of non-pA transcripts.

## Author Summary

Transcription termination is an important regulatory event for both non-coding and coding transcripts. Using high-throughput sequencing, we have mapped RNA Polymerase II's position in the genome after depletion of termination factors from the nucleus. We found that depletion of Ysh1 and Sen1 cause build up of polymerase directly downstream of coding and non-coding genes, respectively. Depletion of Nrd1 causes an increase in polymerase that is distributed up to 1,000 bases downstream of non-coding genes. The depletion of Nrd1 helped us to identify more than 250 unique termination regions for non-coding RNAs. Within this set of newly identified non-coding termination regions, we are further able to classify them based on sequence motif similarities, suggesting a functional role for different terminator motifs. The role of these factors in transcriptional termination of coding and/or non-coding transcripts can be inferred from the effect of polymerase's position downstream of given termination sites. This method of depletion and sequencing can be used to further elucidate other factors whose importance to transcription has yet to be determined.

## Introduction

Termination of RNA Polymerase II (Pol II) transcription plays an essential role in the transcription cycle and has been the subject of several recent reviews [1], [2]. Disruption of the elongation complex at terminators recycles Pol II maintaining a pool of free enzyme able to compete for unoccupied promoters. Correct termination also prevents Pol II from interfering with expression of downstream genes either by colliding with oncoming Pol II elongation complexes or by dislodging transcription factors from the downstream DNA template [3]–[5]. Termination can also serve a regulatory purpose. Several yeast genes are regulated by premature termination [6]–[8] and genes involved in yeast nucleotide metabolism are regulated by the choice of alternative transcription start sites, one of which leads to premature termination [7], [9]. More recent studies have shown that correct termination is also necessary for efficient re-initiation at the same gene through the formation of a loop between the 3′ and 5′ ends [10], [11].

In addition to mRNAs, Pol II transcribes a diverse set of non-coding RNAs including snoRNA, some snRNAs and several classes of ncRNA with unknown functions. In yeast, this pervasive transcription [12]–[14] falls into several classes. Cryptic unstable transcripts (CUTs) are turned over rapidly by the nuclear exosome [15]–[20]. In contrast, another class of yeast ncRNA, termed stable uncharacterized transcripts (SUTs) are observed in the presence of an active nuclear exosome [21].

Pol II terminates coding and non-coding transcripts by different mechanisms [1], [2], [22]–[24]. Coding transcripts and possibly some SUTs are processed at the 3′-end by the cleavage and polyadenylation (pA) machinery. This reaction is coupled to termination occurring downstream of the processing site [1], [2]. In contrast, ncRNAs are terminated and processed by an alternative pathway that, in yeast, requires the RNA-binding proteins Nrd1 and Nab3 and the RNA helicase Sen1 [25]–[29].

Yeast Pol II terminators contain short RNA sequences that bind proteins within large complexes associated with the elongating Pol II. Loosely conserved pA signal sequences downstream of protein-coding genes bind to components of the CF1 complex leading to assembly of the cleavage and polyadenylation machinery [1]. Termination is coupled to cleavage in a manner that has not yet been completely resolved. Non-pA termination components Nrd1 and Nab3 recognize RNA sequence elements downstream of snoRNAs and CUTs [8], [28], [30]–[34] and this leads to the association of a complex that contains the DNA/RNA helicase Sen1 and the nuclear exosome [35]. The mechanism of termination of these ncRNA transcripts has also not yet been determined.

Several possible mechanisms for Pol II termination have been proposed. The “torpedo” model postulates that cleavage at the pA site exposes an uncapped 5′ end on the nascent transcript that acts as a substrate for the 5′→3′ RNA exonuclease Rat1 in yeast or Xrn2 in metazoans [36]–[38]. The exonuclease degrades the nascent transcript and upon reaching the Pol II elongation complex facilitates termination by an unknown mechanism. Another model postulates that an allosteric change in Pol II occurs upon assembly of the pA complex [39]. A member of this complex, Pcf11 has been shown to dismantle an elongation complex in vitro [40], [41] and it is possible that Nrd1 plays a similar role. The DNA/RNA helicase Sen1 interacts with the Pol II CTD [42] and has been proposed to act like the bacterial termination factor rho and track along the nascent transcript and pry off the elongating Pol II [1], [25], [26], [43], [44]. Mutation of Sen1 has been shown to lead to readthrough of both coding and non-coding transcripts in vivo [45] and in vitro can arrest transcription [46].

Part of the uncertainty in delineating termination mechanisms is identifying which factors operate at which terminators in vivo. In this study we have used PAR-CLIP to map Pol II on the yeast genome in living cells [8], [47]–[49] after depletion from the nucleus of components of the different termination pathways [50]. By comparing the Pol II maps with and without nuclear depletion we are able to map the location of termination to narrow regions of the genome downstream of coding and non-coding genes. This approach avoids the complexity of using transcripts to map terminators as the effect of RNA turnover is eliminated. We show here that depletion of Ysh1, the protein that cleaves nascent pre-mRNA transcripts at the pA site, enhances accumulation of Pol II at the 3′-ends of protein-coding genes. In contrast, both Nrd1 and Sen1 depletion lead to readthrough transcription of ncRNAs and our data has allowed us to map the position of non-pA termination facilitated by these factors.

## Results

### Mapping *in vivo* Pol II termination

**Figure 1:**
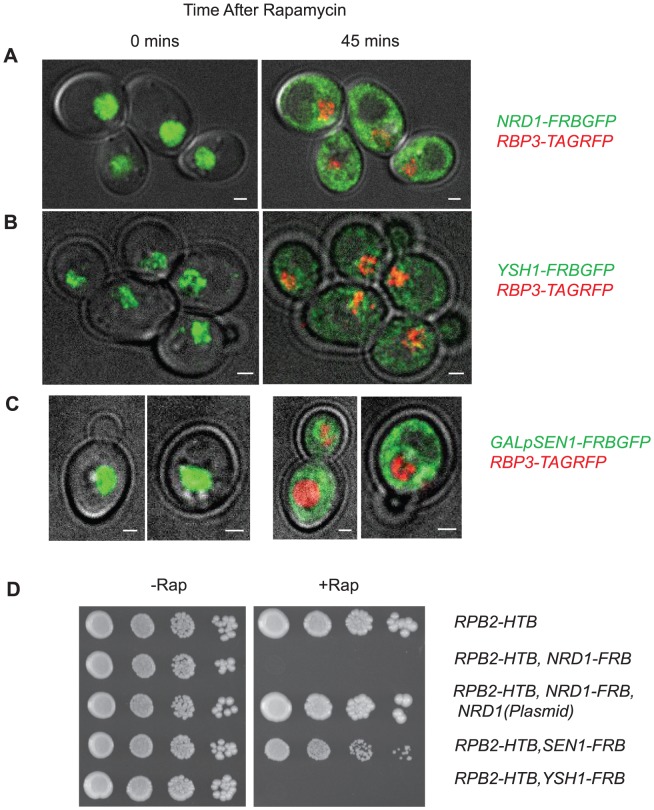
Nuclear depletion of Pol II termination factors. Live yeast cell imaging of: **A.** *NRD1-FRBGFP, RPB3-TAGRFP*; **B.** *YSH1-FRBGFP, RPB3-TAGRFP*; **C.** *GALpSEN1-FRBGFP, RPB3-TAGRFP* strains. Time after rap treatment is indicated above the pictures. **D.** Growth of indicated FRB strains on CSM plates with and without rap.

To map in vivo termination we have used the anchor-away (AA) system [50] to deplete termination factors from the nucleus. In this approach rapamycin (rap) induces a complex between a protein tagged with a FKBP12-rapamycin binding (FRB) domain and an anchor protein tagged with an FKBP12 domain. In our case the FKBP12 domain is on the ribosomal protein *RPL13A* leading to rap-dependent depletion of FRB-tagged protein from the nucleus. Previous work has shown that a Pol II subunit *RPB1-FRB* strain shows greater than 90% depletion of Pol II at the *PMA1* gene within 40 min [51]. We show here that in *NRD1-FRBGFP or YSH1-FRBGFP* strains that also contain, *RPB3-TAGRFP*, Nrd1 and Ysh1 are depleted from the nucleus with similar kinetics (Figure 1A–B). We were unable to observe Sen1-FRBGFP because it is present only in ∼100–2,000 copies per cell. When over-expressed 10–100-fold from a Gal promoter Sen1-FRBGFP is visible and is depleted from the nucleus with similar kinetics (Figure 1C). Given the limitations of background signals in live-cell imaging we cannot rule out the possibility that some small amount of Nrd1, Ysh1 or Sen1 remain in the nucleus. Growth curves show that all three strains grow normally for several divisions after administration of rap and we carried out PAR-CLIP analysis well before any changes in growth were observed (Figure S1A, shaded box). When cells are plated on media containing rap we observe no growth for *YSH1-FRB* and *NRD1-FRB*, while *SEN1-FRB* grows very slowly (Figure 1D).

Growing yeast cultures were cross-linked as previously described [8], [49] with modifications described in Methods. Briefly, 4-thiouracil (4tU) was added to a growing culture for 15 minutes to allow equilibration of uracil pools before the addition of rap. After thirty minutes of rap treatment cultures were irradiated with 365 nm UV for 15 min (Figure S1B). Incubation of yeast cells in 4tU does not significantly affect growth during the course of the experiment as cells continue to grow beyond the time frame of the cross-linking for at least one more doubling (0.2 OD_600_ to 0.7 OD_600_ as measured by BioTek scanner) indicating that 4tU has a less drastic effect on yeast growth than it does in mammalian cells treated for a longer time [52].

To map the position of elongating Pol II we isolated Pol II-bound RNA using a dual 6×His-biotin tagged Pol II Rpb2 subunit (Rpb2-HTB) as previously described [8]. Duplicate libraries were derived from the *RPB2-HTB NRD1-FRB* strain grown in the presence or absence of rap. We also created libraries of Rpb2-bound RNA from *SEN1-FRB* and the parental *RPB2-HTB* strains grown in the presence of rap and a single library from *YSH1-FRB*. Replicate libraries were multiplexed and sequenced by Illumina Hi-seq. Each library yielded between 15–39 million unique reads (Table S1). Biological replicates were strongly correlated (ρ>.99) and thus were added together for analysis (Figure S2). The WT dataset correlates very well to NET-seq [53] and GRO-seq [54] datasets (Figure S3). Thus, PAR-CLIP data accurately represents the position of Pol II elongation complexes on the yeast genome.

### Ysh1-dependent termination

**Figure 2:**
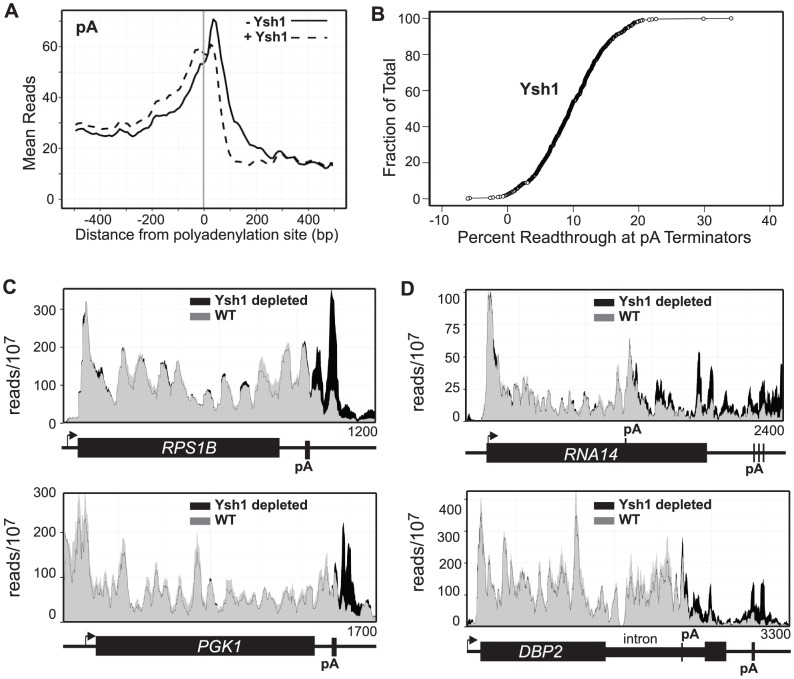
Ysh1 depletion causes readthrough at pA sites. **A.** Mean reads every 10 bp for the 500 most frequently used pA sites with (dotted) and without (solid) Ysh1. **B.** Plot showing percent readthrough of each of 500 pA terminators as a fraction of the total 500 pA terminators. **C and D.** Histograms representing normalized reads with Ysh1 (grey) and without Ysh1 (black) at the given genomic locations. The TSS with the direction of transcription is indicated by an arrow. Genes and pA sites are represented below each graph and the length of the genome depicted is given in the lower right hand corner.

To map pA-dependent termination we carried out PAR-CLIP on Rpb2 after depletion of Ysh1, the factor that has been shown to be required for cleavage of the nascent mRNA at the *GAL7* and *CYC1* pA sites [55]. We reasoned that failure to cleave the nascent transcript would prevent Rat1 degradation thus disabling the “torpedo” mechanism. While we cannot rule out the possibility that other factors are depleted along with Ysh1 we do observe a failure to cleave at the pA site for several genes (Figure S4). The most dramatic effect of Ysh1 depletion is seen in a buildup of reads just downstream of the major pA site of highly expressed protein-coding genes (Figure 2A and 2C). The peak of reads is present in most of the highly expressed protein-coding genes as seen in figure 2B. No similar effect is seen downstream of snoRNAs or CUTS. One surprising aspect of Ysh1 depletion is that readthrough Pol II does not extend further downstream but seems to pause or terminate within 200 bp of the pA site (Figure 2A). This result indicates that Pol II that reads through termination signals fails to elongate but is unable to efficiently terminate.

We do, however, observe several instances of Pol II extending 1 kb or more downstream of apparent terminators in response to Ysh1 depletion. The *RNA14* and *DBP2* genes show an increase of Pol II toward the 3′ end of the coding region indicating the presence of a Ysh1-dependent terminator upstream of the main pA site (Figure 2D). Rna14p is part of the CF1 complex required for recognition of pA sites [1] and previous studies have shown that in addition to the mature 2.2 kb mRNA several smaller mRNAs are present under normal conditions [56], [57]. The terminator we map in *RNA14* is located in the same region as the 3′ end of the shorter (1 kb) mRNA (Figure 2D).

Dbp2p is a DEAD-box RNA helicase that plays a role in assembly of mRNP complexes and in RNA quality control [58], [59]. The *DBP2* gene is unique in yeast in having a long (1 kb) intron localized toward the 3′ end of the coding region and previous work has shown that the gene is autoregulated through sequences in the intron [60]. In Figure 2D we show that depletion of Ysh1 leads to an increase in Pol II cross-linking downstream of the intron suggesting the presence of an upstream terminator. Consistent with this view polyadenylation sites have been mapped to this location in the Dbp2 intron [61]–[63].

### Nrd1-dependent termination

Previous work has shown that Nrd1p is required for proper termination of ncRNAs like snoRNAs and CUTs as well as premature termination of coding transcripts of genes regulated by attenuation [6], [7], [9], [19], [28], [35], [64]. Here we show that depletion of Nrd1 in the presence of rap leads to readthrough transcription of a large number of these ncRNAs. Similar results have recently been obtained by a different protocol [65].

To map the position of termination we used an approach similar to that used to calculate a travelling ratio [66] or Escape Index [38], [65]. Reads in 500 bp windows upstream and downstream of a fixed point were tallied and a ratio of reads in the downstream window to total reads in both windows were calculated for that point. This ratio was determined for each point in the genome on both strands for both control and rap treated cells. Subtracting the fraction of readthrough of the control data from the rap-treated data resulted in a Readthrough Index that allowed us to rank order the regions of the genome showing the highest level of Nrd1-dependent readthrough (Table S2).

**Figure 3:**
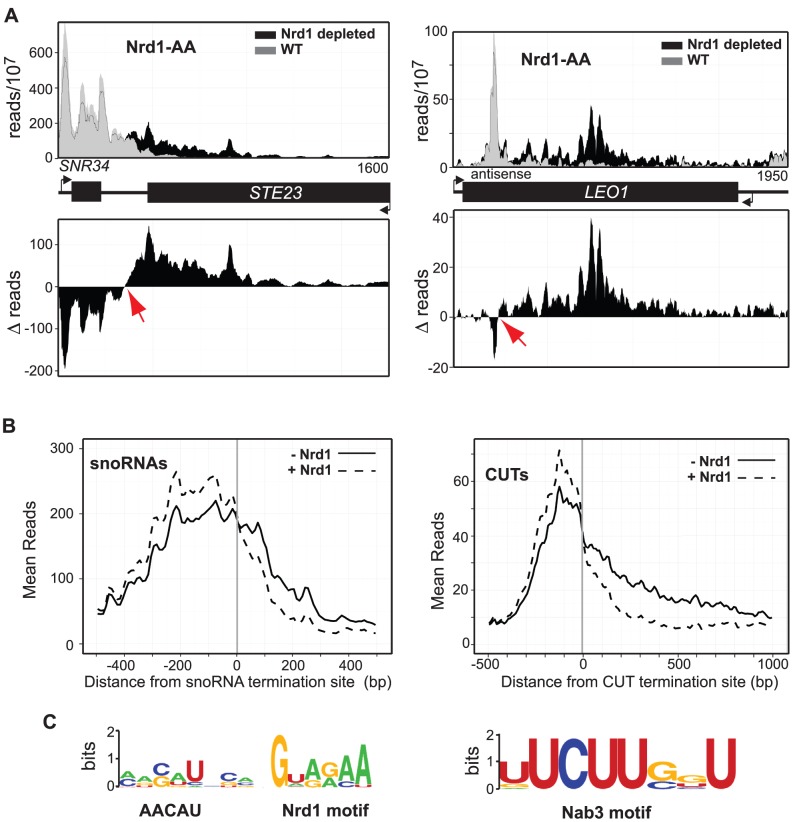
Mapping Nrd1-dependent terminators. **A.** Histograms representing of normalized reads for *NRD1-FRB* with Nrd1 (grey) and without Nrd1 (black) at the given genomic locations. The difference between WT and Nrd1-depleted at every nucleotide is represented as a histogram below each graph. **B.** Mean reads every 10 bp for 49 snoRNA termination sites showing the highest level of readthrough and 144 CUT termination sites with Nrd1 (dotted) and without Nrd1 (solid). **C.** Most abundant Nrd1 motif and the most abundant Nab3 motif in the 150 nt 5′ of the snoRNA and CUT terminators, respectively. MEME input parameters were any number of motifs of length 4–15 nt. Results are presented using WebLogo [92].

Once these regions were identified, we created plots of the difference between the reads in the control and reads with rap (Figure 3A). Nrd1 depletion leads to both a reduction of Pol II upstream and an increase of Poll II downstream of apparent termination sites. This is consistent with previous reports that Pol II pauses prior to termination [67]–[70]. In the absence of Nrd1, this apparent pause is eliminated and Pol II is able to transcribe through the termination site. Fitting this difference plot with a spline function allowed us to determine the point at which the difference plot crosses the X-axis. We have called this the termination site but this represents the center point of a narrow (about 50 nt) region over which Pol II is released from the template.

In Figure 3A we show the difference plots for snoRNA *snR34* and the antisense CUT to the *LEO1* gene demonstrating how the termination sites were identified. We have carried out this analysis for 49 snoRNAs and for 144 CUTs showing the highest level of readthrough transcription (Tables S2 and S3). Comparing these termination sites to the sites determined by Pol II ChIP [65] reveals that 70% of the Schulz et al. sites are more than 100 nt downstream of our termination sites and 25% are more than 200 nt downstream (Figure S5). We do not know the reason for this systematic discrepancy but the PAR-CLIP analysis has greater resolution than Pol II ChIP and unlike ChIP gives strand-specificity. The metagene analysis of the ncRNAs for which we have mapped termination sites is shown in Figure 3B demonstrating a more significant increase in readthrough transcription for Nrd1-depletion when compared to the increase in readthrough transcription due to Ysh1 depletion (Figure 2A). SnoRNA and CUT Nrd1-dependent readthrough extends over 500 bp and 1 kb, respectively.

Examining DNA sequences upstream of the termination site for these sets of snoRNAs and CUTs revealed an interesting difference in the occurrence of Nrd1 and Nab3 binding motifs. The top hit in the MEME analysis [71] of the top 27 snoRNA upstream regions (Figure 3C) include the GUA[A/G] sequence previously identified as a Nrd1 binding site [8], [31]–[34], [72]. This analysis also identifies a loosely conserved sequence AACUA centered about seven nucleotides upstream of the GUA[A/G] sequence that has not previously been reported. The second most significant motif upstream of the snoRNA termination site was the UCUU sequence that binds Nab3, consistent with the presence of a Nrd1-Nab3 heterodimer [29], [31]. In sequences upstream of CUT terminators the most significant hit was UCUUG which contains the previously identified Nab3-binding sequence but with a downstream G as has been observed previously [8], [33], [34]. Among the 144 CUT terminators the Nrd1 binding motif was not present above background. Together, these observations suggest a significant difference in the manner of recognition of snoRNA and CUT terminators.

### U-rich sequences in the Nrd1-dependent termination region

**Figure 4:**
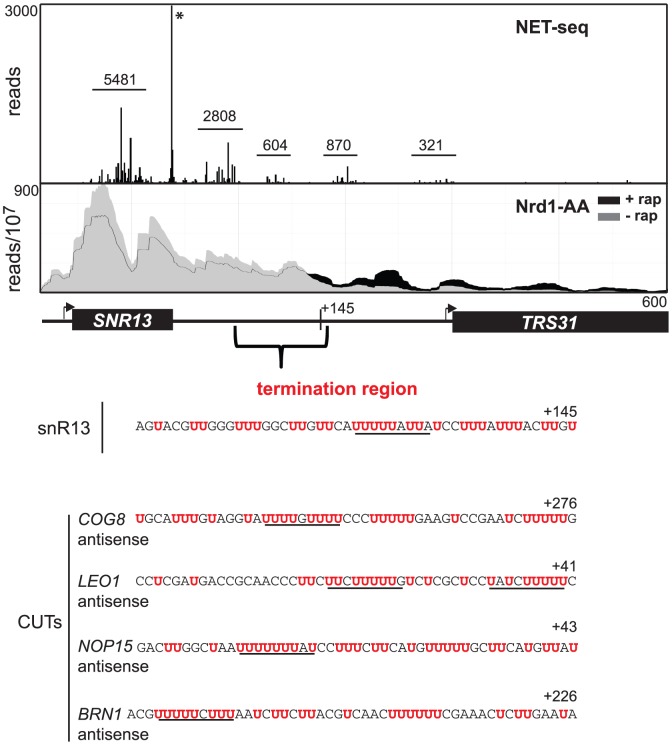
SNR13 termination region. NET-Seq data from Churchman and Weissman [53] are shown above PAR-CLIP data from the *SNR13-TRS31* locus. The number of NET-seq reads from peaks of Pol II are shown above the lines. The asterisk indicates reads derived from mature snR13 RNA that contaminates the NET-seq library. The calculated *SNR13* termination region has been expanded to show U-rich sequences (red) surrounding the termination point. The sequences of several antisense CUT termination regions are shown below the SNR13 sequence.

In Figure 4 we show NET-seq [53] and PAR-CLIP data demonstrating that Pol II levels decline in the *SNR13*-*TRS31* intergenic region. NET-seq reads for several peaks, indicated by bars, are reduced several-fold immediately downstream from the 3′-end of the *SNR13* gene and more than 6-fold further than 100 nt downstream. PAR-CLIP reads from cells grown in the absence of rap decline at the same region reaching a minimum at about 150 nt downstream of the *SNR13* 3′-end. A similar correspondence between NET-seq and PAR-CLIP data is seen for other snoRNA genes (Figure S6). Taken together, these observations indicate that Pol II normally terminates in a Nrd1-dependent fashion in a narrow region located downstream of the snoRNA gene. The sequence from this region for *SNR13*, located from 50–150 nt downstream, is shown below the figure and a series of runs of U residues is indicated in red. This is not unexpected as intergenic regions in *S. cerevisiae* are AT-rich. Sequences of termination regions of several antisense CUTs are shown below the *SNR13* sequence. In these cases we also observe runs of U residues. This is unexpected as these termination regions fall within coding regions (on the opposite strand) which in general are not AT-rich. We propose that these U-rich regions constitute part of the non-poly(A) terminator.

### Nrd1 and RNA turnover

**Figure 5:**
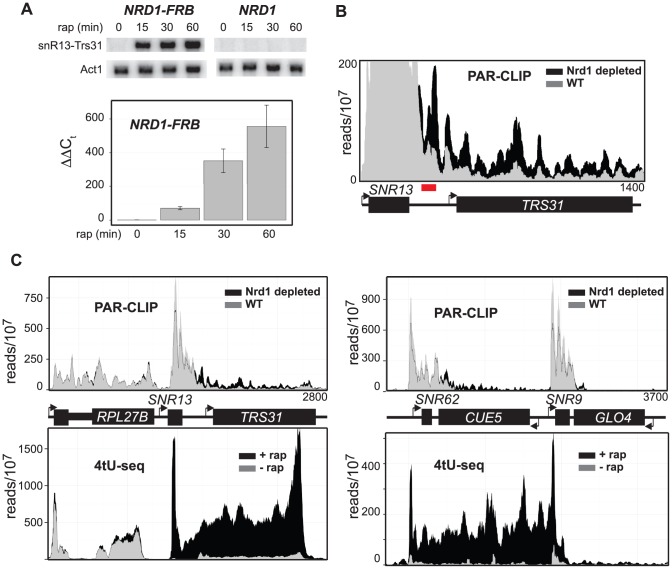
Nrd1 increases the stability of the readthrough transcripts. **A.** Northern blot and qPCR at different times after addition of rap using the amplicon highlighted in red. **B.** Histograms representing normalized reads with Nrd1 (grey) and without Nrd1 (black) at the given genomic locations. The Y-axis has been changed to emphasize the differences between treatment with (black) and without rap (grey) for the *SNR13-TRS31* locus. **C.** Similar to A but Schultz et. al. [65] 4tU-seq data is also represented in as a histogram below each graph for the same region of the genome with Nrd1 (grey) and without Nrd1 (black).

We estimate that *SNR13* readthrough transcripts are present at less than one copy per cell in wild-type cells and this is consistent with the low level of readthrough transcripts seen in the absence of rap in the Northern blot shown in Figure 5A. Despite this low level of steady-state readthrough transcripts we observe significant cross-linking to Rpb2 in the region downstream of *SNR13* (Figure 5B) even in the presence of Nrd1. Presumably the RNA synthesized by these polymerases is rapidly degraded by the nuclear exosome. Figure 5A shows that snR13 readthrough transcripts detected by Northern blot or RT-PCR are increased more than 10-fold by 15 min and more than 100-fold by 30 min of Nrd1 depletion. However, depletion of Nrd1 results in only a 2–3-fold increase in PAR-CLIP signal for Rpb2 (Figure 5B) indicating that depletion of Nrd1 not only allows readthrough of ncRNA transcripts but also results in their stabilization by uncoupling degradation by the nuclear exosome.

Our analysis of steady-state RNA levels is consistent with recently published 4tU-seq data [65]. Figure 5C shows the comparison of our data and that of Schulz et al for *SNR13* and the upstream *RPL27B* gene and for the *SNR62* and *SNR9* genes. 4tU-seq data show a dramatic increase in *SNR13* and *SNR62* readthrough transcription compared to PAR-CLIP data. The most likely explanation for this difference is that 4tU labeling is not completely restricted to nascent transcripts. Labeling cells for six minutes with 4tU allows for multiple rounds of transcription especially for heavily transcribed genes like snoRNAs and ribosomal protein genes. The earliest synthesized transcripts are subject to processing as is clearly evident in the paucity of reads derived from the intron of *RPL27B* in the 4tU-seq compared to the PAR-CLIP (Figure 5C) and a number of other intron-containing genes (Figure S7). Depletion of Nrd1 does not affect all snoRNAs as is seen in Figure 5C where neither PAR-CLIP nor 4tU-seq detect appreciable readthrough transcription at *SNR9*.

### Sen1-dependent termination

**Figure 6:**
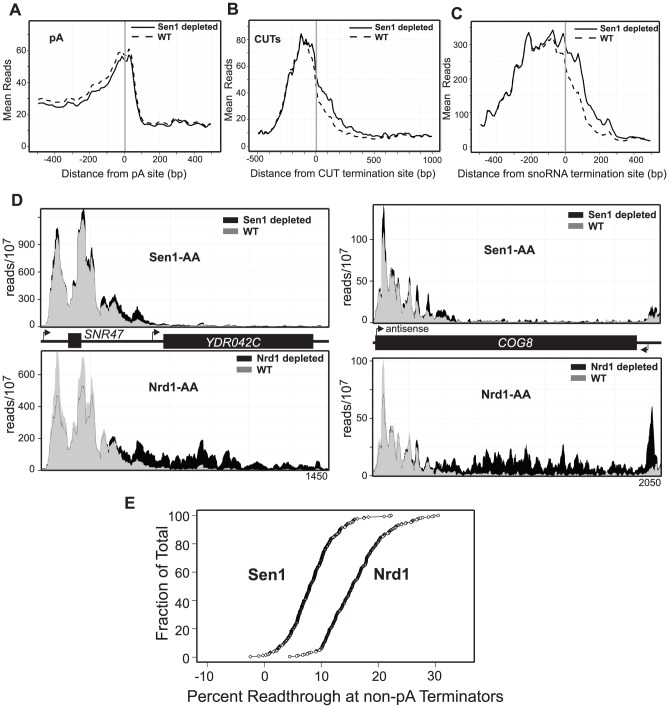
Sen1 depletion causes readthrough at non-pA terminators. **A.–C.** Mean reads every 10 bp for the top 500 polyA,144 calculated CUT termination sites, and 49 snoRNA termination sites, with Sen1 (dotted) and without Sen1 (solid). **D.** Histograms representing normalized reads with Sen1 (grey) and without Sen1 (black) at the given genomic locations. The same region is also represented in as a histogram below each graph with Nrd1 (grey) and without Nrd1 (black). **E.** Percent readthrough of non-pA terminators (ordered from highest to lowest) represented as a fraction of the total number of calculated non-pA terminators. *SEN1-FRB* is represented as diamonds and *NRD1-FRB* is represented as circles.

A role for Sen1p in termination of both coding and ncRNA transcripts has been proposed [26], [28], [45]. Our current data show that depletion of Sen1 results in no change in the localization of Pol II at the 3′-ends of most protein-coding genes (Figure 6A) including those previously shown [45] to be dependent on Sen1 (Figure S8). Thus, despite our previous observation that Sen1 cross-links to these transcripts [8], there is little evidence for a role for Sen1 in pA termination. Sen1 depletion does result in readthrough transcription at both snoRNAs and CUTS (Figure 6B–C) which is consistent with previous observations [15], [19], [28]. The pattern of Pol II readthrough in response to Sen1 depletion differs, however, from that of Nrd1. Rather than an extended region of readthrough downstream of the terminator, Sen1 depletion often results in an increase just downstream by a few hundred nucleotides. This type of pattern is seen in Figure 6D for *SNR47* and the CUT antisense to the *COG8* gene and in Figure S9 for several protein-coding genes regulated by Nrd1-dependent attenuation. Figure 6E compares the percent readthrough downstream of all 266 non-pA terminators in Sen1 depleted and Nrd1 depleted cells. The percent readthrough after Nrd1 depletion is twice that for Sen1 depletion and these values are not dominated by a subset of terminators. This observation suggests that Sen1 may play a different function than Nrd1 at these genes and that loss of Sen1 results in less processive or more pause prone readthrough transcription.

## Discussion

In this paper we describe the development and implementation of a technique for mapping the position of in vivo Pol II termination. Depletion of components of the yeast Pol II termination machinery followed by Pol II cross-linking to nascent RNA has allowed the identification of both pA and non-pA termination regions. The site of pA termination has been mapped to within a few hundred bases of the pA site as has previously been observed for the Cyc1 terminator [73]. We have identified non-pA terminators using an algorithm that identifies points in the genome where Pol II readthrough increases in response to Nrd1 depletion.

While many of the components of both the pA and non-pA termination complexes have been identified, the mechanism of termination is still unclear. Two general models have been proposed [1], [2], [22], [74]. The allosteric model proposes a conformational change in Pol II that slows elongation [39], [75], [76] while the torpedo model proposes that cleavage at the pA site creates a substrate for a 5′→3′ exonuclease (Rat1 in yeast and Xrn2 in metazoa) that degrades the nascent transcript like a “fuse” and upon reaching elongating Pol II somehow facilitate termination [36], [37], [77]. Neither model adequately explains all experimental observations leading to a hybrid model that includes both allosteric and topedo mechanisms [78].

### pA termination

Our data on Ysh1 depletion supports a two-step pause and release model for termination that invokes both allosteric and torpedo mechanisms [79]. The buildup of Pol II just downstream of the uncleaved pA site suggests that Pol II has paused but is deficient in the subsequent release step. This is consistent with a role for Ysh1 in providing the substrate for the Rat1 exonuclease that facilitates removal of Pol II from the template [80]. Ysh1 apparently is not required for the allosteric change that results in pausing downstream of the pA site as seen in the lack of readthrough at most pA sites. In contrast, mutation of the pA site [81] or mutations in *RNA14*, *RNA15*, *PCF11*, *CLP1* and *GLC7* result in readthrough transcription more than 500 nt downstream [73], [82], [83] arguing for a role for these factors in the allosteric change that renders Pol II immobilized.

Depletion of Ysh1 has also revealed several genes that are apparently regulated by premature termination through the pA pathway. *RNA14* and *DBP2* contain pA sites upstream from the 3′ pA site. Loss of Ysh1 leads to processive elongation ending at the downstream pA site indicating that Ysh1 is required for termination at these upstream sites. Why the putative allosteric step does not function efficiently at these upstream sites is not clear. Perhaps the phosphorylation pattern of the CTD at these sites precludes the assembly of factors like Pcf11. Alternatively, chromatin structure modification induced by the Pol II that elongates through these terminators may suppress normal termination. Finally, it is possible that use of the downstream terminator by Pol II that ignores the upstream pA site creates a gene loop that favors termination at the downstream site.

### Non-pA termination

Nrd1 and Nab3 bind specific RNA sequences and act as sensors to detect non-pA terminator sequences in the nascent transcript. While a number of studies have characterized the short motifs recognized by Nrd1 and Nab3 the relative orientation and abundance of these sequences varies widely among non-pA terminators [8], [31]–[34], [49], [65]. In this study we have localized termination downstream of 49 snoRNAs and 144 CUT transcripts. This has allowed a search for sequences that may define these two sets of terminators. We find that the most significant motif in this set of snoRNA terminators contains the GUA[A/G] motif previously identified for Nrd1 in the context of a longer sequence that indicates the possible involvement of another, unidentified RNA-binding protein. The second most significant motif is the Nab3 binding sequence UCUUG. In the case of the CUT terminators the only significant motif is the Nab3 binding sequence. Thus, it appears that while both Nrd1 and Nab3 binding contribute to recognition of snoRNA terminators Nab3 predominates for CUTs. This does not preclude Nrd1 from playing a critical role in termination at these CUTs. We have previously shown that Nrd1 is necessary for termination of the CUT antisense to *FMP40* presumably by acting as an adaptor to couple the Nrd1-Nab3-Sen1 complex to the elongating Pol II through interaction between the Nrd1 CID and the CTD [15].

No other significant motifs were observed upstream of these terminators but we did observe that the sequences surrounding the termination site are U-rich and contain multiple runs of U residues. This is not unexpected for snoRNA downstream sequences as intergenic regions are AT-rich in *S. cerevisiae*. The U-rich sequences surrounding the termination sites (Figure 4) of antisense CUTs are more significant as these sequences occur in the context of a coding region on the opposite strand. U-rich sequences have been shown to form unstable rU:dA base pairs [84] and in the hybrid binding site of Pol II transcribing the antisense CUT such an unstable hybrid sequence may increase the probability of termination [85], [86].

In addition to its role in Pol II termination, our data highlight the role Nrd1 plays in the turnover of ncRNAs. Nrd1 depletion by anchor away leads to accumulation of readthrough transcripts in 4tU-seq to a much higher degree than observed by our Rpb2-HTB PAR-CLIP. We show here by Northern and RT-PCR that after 40 min of Nrd1 depletion the level of snR13 readthrough transcripts increases over 100-fold. However, after 40 minutes of depletion the amount of readthrough as observed by PAR-CLIP is less than 10-fold. This difference is likely due to an important role for Nrd1 in coupling termination to turnover of the completed transcript. Nrd1 is found in a complex with components of the TRAMP and exosome complexes [35] and previous studies have indicated that *nrd1* mutants are deficient in RNA turnover [15], [19], [35].

Pulse labeling RNA with 4tU was recently used to analyze newly synthesized transcripts in a Nrd1 anchor away mutant and this data identified over 1500 Nrd1-dependent transcripts [65], far more than the number of terminators we describe here. Part of the reason for this discrepancy is that the six-minute pulse used in the 4tU-seq protocol is substantially longer than the time needed to synthesize short RNAs. Thus, 4tU-seq over-estimates the effect on termination and may ascribe termination functions to cases where the role in turnover is independent of termination.

Depletion of the DNA/RNA helicase Sen1 has allowed us to place this factor squarely in the Nrd1-Nab3 non-pA termination pathway. We observe readthrough transcription downstream of both snoRNAs and CUTs but not downstream of pA sites. Pol II does not progress far downstream after Sen1 depletion indicating that this factor may act after the allosteric change has occurred. This is consistent with Sen1 acting in place of Rat1 to provide the activity that dislodges the paused Pol II [46] and supports previous work showing the difference in the requirement for these factors in pA and non-pA termination [24].

**Figure 7:**
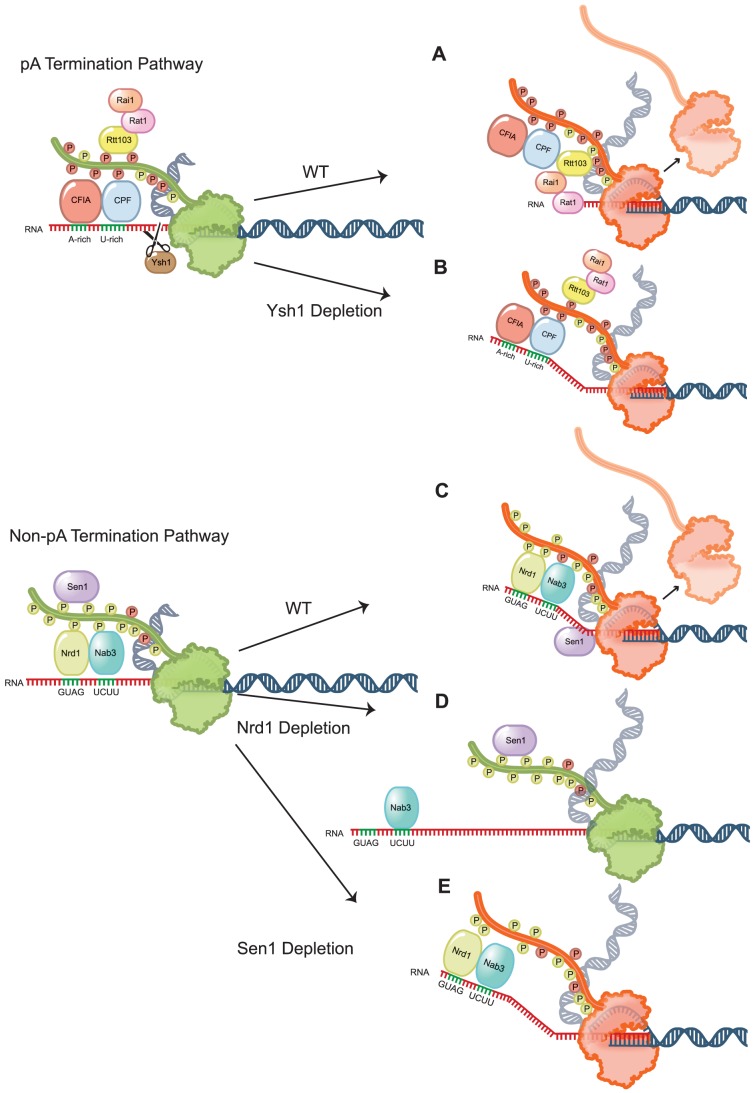
Schematic representation of Pol II termination after removal of non-pA and pA termination factors. Elongating Pol II (green) terminates pA transcripts (A) after an allosteric change (red) that reduces processivity. (B) Depletion of Ysh1 leads to minimally extended readthrough transcripts but does not block the allosteric change in Pol II. (C) Nrd1 and Nab3 binding recruit Sen1 for termination of non-pA transcripts. (D) Pol II elongation complex lacking Nrd1 does not recognize termination sequences in the nascent transcript and thus does not facilitate the allosteric transition in Pol II. This leads to processive readthrough. (E) Nrd1 and Nab3 recognize terminator sequences allowing the allosteric change in Pol II but depletion of Sen1 blocks removal of Pol II from the template.

The results presented here are summarized in a model shown in Figure 7. We have been able to demonstrate a difference in the global effect of depleting different components of the yeast termination machinery. These results have been interpreted in the context of a two state model for termination in which terminator sequences in the nascent RNA trigger assembly of protein complexes that signal to the elongating Pol II to decrease its elongation rate and processivity. These anti-processivity factors include Pcf11 for pA terminators and Nrd1 for non-pA terminators. Loss of either of these factors leads to runaway processive elongation. In contrast, depletion of Ysh1 or Sen1 does not block the transition to non-processive elongation but does result in a defect in removal of Pol II from the template. For Sen1 this likely occurs through loss of a rho-like function [44], [46] while Ysh1 depletion leads to a lack of cleavage at the pA site thereby limiting access of Rat1 and preventing the torpedo mechanism of Pol II release [80].

The data presented here demonstrate that PAR-CLIP analysis of Pol II in living yeast cells can define the site of Pol II termination regions and when coupled with depletion of termination factors offers an avenue for examining the roles of different factors in termination genome-wide. Such experiments will illuminate the variety of mechanisms the cell uses to prevent Pol II from transcribing beyond genetically determined 3′ boundaries.

## Materials and Methods

### Strain construction

Yeast strains for anchor away were constructed using the parental strain HHY168 [50] (Euroscarf #Y40343). *RPB2* was C-terminally tagged with an HTB (6×HIS, TEV, and Biotin) tag as described previously [8], [87]. *NRD1, YSH1, and SEN1* were C-terminally tagged with FRB and FRBGFP as described in Haruki et al. [50]. The SEN1 promotor was replaced with the *GAL1* promotor using a cassette from pFA6a-kanMX6-PGAL1-HBH [87]. All strains used in these experiments are listed in Table S4.

### Cell growth

Cells were grown in Complete Synthetic Media (CSM) supplemented with 2% glucose or galactose and 40 mg/l adenine. For RNA analysis, cells were seeded to an OD_600_ of 0.1 from a 5 ml or 50 ml overnight culture. The cells were then incubated at 30°C to an OD_600_ of 1. 1 µg/ml of rap (LC laboratories) was then added to the cultures and the cells were allowed to grow for the indicated times in the experiment. For growth curves, a BioTek Infinity 2 (BioTek) was used to incubate a 24 well plate containing CSM media with and without 1 µg/ml rap (added after 6 hrs of growth) with orbital shaking (Slow, 1 mm amplitude). Cells were diluted to an OD_600_ of 0.1 per well and an OD_600_ was measured every 10 min for 24 hours.

### RNA purification and cDNA synthesis

Total RNA was extracted from yeast with hot acid phenol as previously described [8]. Strand specific northern blots were done as described previously [88] with the following modifications. Ultrahyb (Invitrogen) was used instead of the described hybridization buffer. Real-time PCR analysis was done on a CFX96 instrument (Biorad) in triplicate as described previously [49]. Primers used can be found in Table S5.

### Live cell imaging of FRBGFP strains

The strains containing *NRD1-FRBGFP, YSH1-FRBGFP, or GALpSEN1-FRBGFP, RPB3-TAGRFP* were grown to an OD_600_ of 1 at 30°C. Live cell imaging was done on a Deltavision microscope (Applied Precision) as described previously [89] with the following modification: 1 µg/ml rapamyacin was added to the agarose/media pad. Galactose was substituted for glucose with the GALpSEN1-FRBGFP strain. Pictures were taken of GFP at 0 mins and 45 mins post rap exposure and 45 mins for RFP.

### Anchor away and PAR-CLIP

Cells were grown overnight 30°C in 50 ml of YPD (Yeast Extract Peptone with 2% glucose) media. Four 500 ml flasks of sterile cross-linking media (CSM–Ura supplemented with 2% glucose, 40 mg/l adenine, 60 µM uracil, and 1 µM biotin were seeded to an OD_600_ of 0.1 and incubated at 30°C until an OD_600_ of 1. Crosslinking was carried out by the protocol described in Figure S1B. 4-thiouracil (4tU) was added to a final concentration of 4 mM and incubated at 30°C for 15 min. 1 µg/ml of rapamycin was then added to the cells and incubated at 30°C for 30 min. All four 500 ml cultures were then pooled into one 2 l beaker and irradiated from a distance of two cm with 365 nm UV (∼1 W/cm^2^) from an LC-L5 LED UV Lamp (Hamamatsu) for 15 min with gentle stirring. The top ∼2 mm of the culture appears luminescent indicating that the UV light does not penetrate further into the culture. Assuming an even mixture of the culture we calculate that the average cell is cross-linked for only ∼10 seconds within the 15 min crosslinking period. The cultures were then filtered through 0.45 micron nitrocellulose filters (Millipore) and cells were scraped into 5 ml Buffer 1 (300 mM NaCl, 0.5% NP-40, 50 mM NaPO_4_ pH 7.2, 10 mM immidazole, 6 M Guanidine HCl, Protease inhibitor Cocktail VII [RPI]) and frozen in liquid nitrogen.

HTB tagged Rpb2 purification was adapted from our previously published protocol [49]. Cells were lysed in liquid nitrogen using a SamplePrep 6870 freezer mill (Spex) with 15 cycles per second of cracking for 15 cycles of 1 min with 2 min of cooling between each cycle. Lysates were then incubated with 1 ml of Ni-NTA agarose (Qiagen), which was equilibrated in buffer 1 for 2 hours at room temperature. The Ni-NTA agarose was then added to an empty 10 mL plastic column (Biorad) and washed with 20 ml of Buffer 1 followed by 10 ml of Buffer 2 (20 mM NaPO_4_ pH 7.2, 300 mM NaCl, 0.5% NP40, 10 mM imidazole, 4 M Urea). The protein was eluted off of the Ni-NTA agarose with 5 ml Buffer 2+250 mM immidazole and into 5 ml Buffer 2+120 µl Protease inhibitor Cocktail VII+100 µl of hydrophilic streptavidin magnetic beads (NEB). The slurry was allowed to incubate for 4 hours at room temperature. The strepavadin magnetic beads were then resuspended in 500 ul of Buffer 3 (50 mM Tris pH 7.4, 200 mM NaCl, 4 M Urea), transferred to a 1.5 ml siliconized tube and washed with 3×1 ml of Buffer 3 followed by 3×1 ml of T1 Buffer (50 mM Tris pH 7.4, 150 mM NaCl, 2 mM EDTA). Beads were resuspended in 200 µl of T1 buffer. 0.15 U/µl of Rnase T1 (Fermentas) was added to the bead slurry and allowed to incubate at 25°C for 15 min. The beads were then washed 3×1 ml T1 wash buffer (50 mM Tris pH 7.4, 500 mM NaCl, 1% NP-40, 0.5% Na deoxycholate) followed by 3×1 ml washes in PNK Buffer (50 mM Tris pH 7.2, 50 mM NaCl, 10 mM MgCl_2_). The beads were resuspended in 200 µl of TSAP reaction solution (0.15 U/ul Thermosensitive Alkaline Phosphatase [TSAP, Promega], 1 U/µl SupeRase Inhibitor [Invitrogen], 1 mM DTT in PNK Buffer) and incubated at 37°C for 30 min. The beads were washed 1×1 ml Buffer 3, 2×1 ml T1 Wash Buffer, and 3×1 ml PNK Buffer.

### cDNA library preparation

Streptavadin beads were resuspended in 200 ul of PNK reaction solution (1 U/µl T4 PNK [NEB], ^32^P γ-ATP, 5 mM DTT in PNK Buffer) and incubated at 37°C for 30 min. 1 mM of cold ATP was added to the reaction and incubated at 37°C for 10 min. The beads were washed 1×1 ml Buffer 3, 1×1 ml T1 wash, 3×1 ml T4 RNA Ligase Buffer (50 mM Tris pH 7.4, 10 mM MgCl_2_). The beads were resuspended in 44 µl of T4 RNA Ligase 2 reaction solution (25% PEG 8000, 5 µM 3′ adaptor [AppAGATCGGAAGAGCACACGTCTddC, IDT], 10 U/µl T4 RNA Ligase 2, truncated K227Q [NEB], 2 U/µl RNase Inhibitor [Invitrogen], 1 mM DTT in RNA Ligase Buffer) and incubated at 25°C for 4 hours. The beads were then washed with 1×1 ml Buffer 3 and 5×1 ml T4 RNA Ligase Buffer. The beads were resuspended in 50 ul of T4 RNA Ligase reaction solution (5 uM 5′ Adaptor [GUUCAGAGUUCUACAGUCCGACGAUC, IDT], 1.2 U/µl RNase Inhibitor, 1 mM ATP, 0.5 U/µl T4 RNA Ligase [NEB], 1 mM DTT in RNA Ligase Buffer) and incubated at 16°C overnight. The beads were then washed 5×1 ml Proteinase K Buffer (100 mM Tris pH 7.4, 150 mM NaCl, 12.5 mM EDTA). The beads were resuspended in 200 µl of Proteinase K Buffer +2% SDS and 12 µl of Proteinase K (NEB). The suspension was incubated for 30 min at 37°C at which point the supernatant was removed and saved and the beads were resuspended in 200 µl of Proteinase K Buffer +2% SDS and 12 µl of Proteinase K. The beads were then incubated 30 min at 37°C and the supernatants were pooled. The RNA was recovered by acid phenol/chloroform extraction followed by two serial ethanol precipitations. The resulting pellet was allowed to dry and resuspended in 15 µl of Nuclease-Free Water [Invitrogen]. The RNA was split into 5 µL aliquots and frozen at −80°C for storage.

The Reverse Transcription, PCR, and gel extraction of the cDNA Library were carried out as described previously [49] with the following modifications. For reverse transcription, the primer used was AGACGTGTGCTCTTCCGATCT (IDT). For PCR analysis, biological repeats were multiplexed with the following primers. The forward primer was AATGATACGGCGACCACCGAGATCTACACGTTGAGAGTTCTACAGTCCG*A (where a * denotes and phosphorothioate bond). The first reverse primer was CAAGCAGAAGACGGCATACGAGATATTGGCGTGACTGGAGTTCAGACGTGTGCTCTTCGGATC*T for the first biological repeat and the second reverse primer was CAAGCAGAAGACGGCATACGAGATTACAAGGTGACTGGAGTTCAGACGTGTGCTCTTCCGATC*T for the second biological repeat (IDT).

### Sequencing and bioinformatics

Sequencing and demultiplexing was done at UC Riverside on an Illumina HiSeq. Trimming of the resulting sequences as described previously [49]. Briefly, raw reads were trimmed of the 3′ adaptor using a wrapper for R-bioconductor [90] developed by Sarah Wheelan. Raw trimmed reads were condensed (defined as no more than one of the same exact sequence) to eliminate any PCR artifacts within the data. Bowtie 1.0.0 [91] was run with the following arguments (-y –best -v 2) and aligned to the SacCer3 (R64) genome. Reads mapping to tRNA genes were removed as these likely represent artifactual binding during the affinity purification steps. Reads were then converted to a wig format where each read was multiplied by a factor that was defined as the total number of reads aligned per 10^7^ reads. PAR-CLIP datasets have been submitted to GEO with the accession number GSE56435.

### Calculation of the top 500 polyA sites

To calculate the best polyA sites per gene we used the sites provided by Moqtaderi et al. [63]. We set a hard cutoff of at least 200 raw reads. If, however, there were multiple very strong polyA sites within 200 base pairs of each other, we calculated the read ratio between the two most significant polyA sites. If the read ratio was less than 0.4, we ignored them.

### Global readthrough percentage

Global readthrough percentage was calculated for each point in the genome as the percentage readthrough (reads downstream 500 bp/reads downstream 500 bp + reads upstream 500 bp) in the treated sample minus control sample. All points with a percentage readthrough of greater than 10%, at least a total of 1000 reads in either sample, and greater reads in the treatment vs the control were extracted. The list was further refined by taking the difference of every point 2 kb around non-overlapping termination sites (approx 500 total), graphing the region, and then fitting a spline function over a 25 bp window to find the best point of inflection. These points of inflection were then used as a focal point for the meta gene analysis.

## Supporting Information

Figure S1 **A.** Growth of indicated FRB strains in CSM media with and without rap. OD_600_ was taken every 10 min for 20 hours. The grey bar represents the timeline of the experiment. **B.** Timeline of the PAR-CLIP protocol.
(EPS)

Figure S2 **A–D.** Replicate pair-wise comparison of number of reads corresponding to each gene in our PAR-CLIP biological replicates. Spearman coefficient is represented by rho.
(EPS)

Figure S3 **A–C.** Pair wise comparison of number of Rpb2-HTP cross-linked reads in each annotated gene vs. GRO-seq reads [54] or NET-seq reads [53]. Spearman coefficient is represented by rho.
(EPS)

Figure S4 **A–D.** Histograms representing normalized reads with Ysh1 (grey) and without Ysh1 (black) at the given genomic locations. Genes and pA sites are represented below each graph and the length of the genome depicted is given in the lower right hand corner. Below are qPCR at different times after addition of rap using the amplicon highlighted in red.
(EPS)

Figure S5 Relative position on Schulz et al. termination points compared to our PAR-CLIP determined inflection points.
(EPS)

Figure S6 A comparison of NET-Seq data from Churchman and Weissman [53] to our normalized reads at the SNR3 and SNR34 locus.
(EPS)

Figure S7 Comparison of PAR-CLIP data to Schulz et. al. 4tU-seq data [65]. Number of reads for each base pair at the given locus is represented by a histogram. Genes and introns are represented under each graph.
(EPS)

Figure S8 Histograms representing normalized reads with Sen1 (grey) and without Sen1 (black) at the given genomic locations.
(EPS)

Figure S9 Histograms representing normalized reads with Nrd1 (grey) and without Nrd1 (black) for NRD1 and TYE7. The same region is also represented in as a histogram below each graph with Sen1 (grey) and without Sen1 (black).
(EPS)

Table S1 Read counts at various stages of bioinformatic manipulation.
(EPS)

Table S2 Top 266 points of inflection after spline function refinement. CUT annotations are defined in Neil et. al. [18]. NUT annotations are defined in Schulz et. al. [65]. Gene and snoRNA annotations come from the Saccharomyces genome database. Top 144 CUTs highlighted in green. NA is no annotation.
(PDF)

Table S3 Top 49 points of inflection 3′ of snoRNAs after spline function refinement. snoRNA annotations come from the saccharomyces genome database.
(EPS)

Table S4 Yeast strains used in this study.
(EPS)

Table S5 Primers used in this study.
(EPS)
