# Supplementary material for: Genome-Wide Mapping of Yeast RNA Polymerase II Termination
Source: PLoS Genet. 2014 Oct 9;10(10):e1004632. doi: 10.1371/journal.pgen.1004632 (PMC4191890; doi:10.1371/journal.pgen.1004632)
Supplement: Table S2 — Top 266 points of inflection after spline function refinement. CUT annotations are defined in Neil et. al. [18]. NUT annotations are defined in Schulz et. al. [65]. Gene and snoRNA annotations come from the Saccharomyces genome database. Top 144 CUTs highlighted in green. NA is no annotation. (PDF) [file pgen.1004632.s011.pdf]

Name	Chromosome	Inflection Point	Strand	Percentage Readthrough
CUT382	chrXV	553216	+	43.35
CUT721	chrXI	493135	-	40.80
CUT532	chrIV	1300554	-	39.82
NUT0165	chrIV	1365872	+	38.28
CUT285	chrXIII	130024	+	38.02
CUT088	chrIV	1240442	+	36.75
CUT301	chrXIII	535192	+	34.15
SNR65	chrIII	177261	+	34.10
CUT776	chrXIII	545526	-	33.50
CUT881	chrXVI	117824	-	32.08
CUT160	chrVII	884685	+	31.89
CUT710	chrXI	274705	-	31.84
CUT515	chrIV	892127	-	31.41
CUT110	chrV	262699	+	30.59
CUT216	chrX	582732	+	30.10
CUT075	chrIV	974762	+	29.77
NUT0377	chrX	41790	+	29.51
CUT029	chrII	576557	+	29.13
CUT144	chrVII	484779	+	28.88
CUT645	chrVIII	238387	-	28.61
CUT711	chrXI	286132	-	28.45
NUT0410	chrX	466837	+	28.18
CUT804	chrXIV	185131	-	28.16
CUT620	chrVII	762007	-	27.77
CUT300	chrXIII	501221	+	27.67
CUT114	chrV	348057	+	27.62
CUT335	chrXIV	417868	+	27.51
CUT650	chrVIII	381120	-	27.30
CUT827	chrXV	167567	-	27.00
CUT443	chrII	42768	-	26.68
CUT428	chrXVI	676079	+	26.66
CUT422	chrXVI	475789	+	26.56
CUT913	chrXVI	759202	-	26.55
CUT453	chrII	408165	-	26.53
CUT303	chrXIII	557655	+	26.48
CUT596	chrVII	337383	-	26.40
CUT695	chrX	535071	-	26.38
CUT175	chrVIII	171066	+	26.15
CUT249	chrXII	185193	+	26.08
CUT035	chrII	698509	+	25.96
CUT133	chrVII	229364	+	25.88
CUT271	chrXII	738386	+	25.82
NUT0217	chrV	563133	+	25.80
CUT720	chrXI	387150	-	25.58
NUT1393	chrXV	127850	-	25.51
CUT612	chrVII	667501	-	25.29
CUT723	chrXI	554354	-	25.26
NUT0482	chrXII	66941	+	25.13

CUT600	chrVII	412769	-	25.00
CUT507	chrIV	703891	-	24.95
CUT808	chrXIV	359108	-	24.93
NUT0447	chrXI	165231	+	24.88
CUT189	chrIX	203808	+	24.82
CUT232	chrXI	335622	+	24.81
NUT0245	chrVII	79377	+	24.57
NUT0828	chrI	224782	-	24.46
CUT825	chrXV	146911	-	24.37
NUT1010	chrVII	94513	-	24.31
CUT396	chrXV	1001332	+	24.23
NUT1273	chrXII	855366	-	24.18
SNR56	chrII	88276	+	23.99
CUT134	chrVII	269569	+	23.99
NRD1	chrXIV	174307	-	23.95
CUT817	chrXIV	529360	-	23.92
CUT781	chrXIII	646742	-	23.81
CUT109	chrV	238837	+	23.70
NUT0473	chrXI	576393	+	23.47
CUT739	chrXII	505509	-	23.24
CUT011	chrII	306412	+	23.18
CUT542	chrV	15700	-	22.95
CUT615	chrVII	702270	-	22.92
NUT1284	chrXIII	46247	-	22.80
SNR60	chrX	349046	-	22.53
CUT363	chrXV	204881	+	22.37
CUT002	chrI	138767	+	22.23
CUT919	chrXVI	874679	-	22.17
CUT742	chrXII	657775	-	22.16
SNR47	chrIV	541515	-	22.14
NUT1194	chrX	611827	-	22.07
NUT0685	chrXV	136420	+	22.07
CUT472	chrIV	99170	-	22.05
CUT241	chrXI	490117	+	21.94
CUT699	chrX	650898	-	21.83
NA	chrXI	142916	-	21.82
NUT0422	chrX	620770	+	21.78
CUT207	chrX	186970	+	21.78
NUT0344	chrVIII	294313	+	21.68
CUT655	chrVIII	450388	-	21.63
CUT095	chrIV	1359350	+	21.24
CUT415	chrXVI	352836	+	21.15
CUT880	chrXVI	108077	-	21.09
CUT019	chrII	366035	+	21.09
NUT0472	chrXI	566363	+	20.97
CUT319	chrXIV	168487	+	20.90
CUT536	chrIV	1436562	-	20.79
CUT704	chrXI	96084	-	20.71
NUT1351	chrXIV	355951	-	20.69

NUT0728	chrXV	617357	+	20.47
CUT566	chrV	416334	-	20.46
CUT082	chrIV	1149859	+	20.45
CUT332	chrXIV	349044	+	20.26
CUT112	chrV	326283	+	19.92
CUT653	chrVIII	441765	-	19.86
CUT439	chrI	35789	-	19.82
CUT724	chrXI	557207	-	19.81
CUT865	chrXV	849041	-	19.67
CUT617	chrVII	739472	-	19.66
CUT225	chrXI	31140	+	19.40
CUT813	chrXIV	459163	-	19.35
NA	chrXIII	751946	+	19.32
NUT0906	chrIV	599243	-	19.25
CUT505	chrIV	690295	-	19.09
CUT067	chrIV	588279	+	19.04
CUT669	chrIX	381386	-	19.03
CUT701	chrXI	24572	-	18.99
ARO4	chrII	717921	-	18.97
MSG5	chrXIV	530104	+	18.88
CUT081	chrIV	1106256	+	18.86
SNR45	chrXVI	822033	+	18.86
CUT575	chrVI	118993	-	18.82
NUT0645	chrXIV	307589	+	18.81
CUT049	chrIV	57222	+	18.69
CUT113	chrV	343708	+	18.63
CUT883	chrXVI	131958	-	18.62
SNR49	chrXIV	716354	+	18.60
CUT673	chrX	99146	-	18.56
CUT098	chrIV	1427703	+	18.48
CUT065	chrIV	542766	+	18.46
NUT0846	chrII	533166	-	18.40
NA	chrII	235801	-	18.37
MTD1	chrXI	590459	+	18.17
NUT0521	chrXII	500843	+	18.14
SNR34	chrXII	899484	+	18.13
NUT0097	chrIV	264737	+	18.13
NUT0352	chrVIII	461842	+	18.11
CUT092	chrIV	1319619	+	18.06
SNR4	chrV	425029	+	18.04
CUT270	chrXII	721871	+	17.92
CUT806	chrXIV	275679	-	17.92
NUT1180	chrX	380653	-	17.89
NUT0869	chrIII	310523	-	17.82
CUT278	chrXII	1042064	+	17.82
NUT0971	chrV	251351	-	17.76
NA	chrXII	827893	-	17.70
NUT0511	chrXII	388840	+	17.70
NUT1125	chrVIII	551396	-	17.68

NUT0826	chrI	184550	-	17.47
CUT267	chrXII	675014	+	17.46
CUT607	chrVII	576304	-	17.43
NA	chrVII	974893	+	17.42
CUT420	chrXVI	454235	+	17.40
SNR46	chrVII	545607	+	17.31
SNR11	chrXIII	652536	+	17.25
SNR87	chrXI	431085	-	17.24
CUT545	chrV	59103	-	17.15
CUT475	chrIV	134699	-	17.15
NUT0379	chrX	74270	+	17.12
NUT0451	chrXI	200721	+	17.04
CUT869	chrXV	953672	-	16.98
SNR62	chrXV	409626	-	16.91
NUT0356	chrVIII	554353	+	16.90
NUT0288	chrVII	767149	+	16.87
CUT587	chrVII	131130	-	16.79
NUT0107	chrIV	387686	+	16.75
CUT405	chrXVI	170206	+	16.73
SNR8	chrXV	832515	+	16.65
SNR35	chrXV	759204	-	16.58
SNR32	chrVIII	381790	+	16.57
CUT259	chrXII	404950	+	16.47
CUT705	chrXI	158166	-	16.44
CUT183	chrVIII	501773	+	16.41
CUT528	chrIV	1269536	-	16.40
CUT188	chrIX	188137	+	16.33
CUT213	chrX	413916	+	16.29
CUT550	chrV	178282	-	16.26
NUT0532	chrXII	645536	+	16.23
NUT0893	chrIV	365847	-	16.18
CUT805	chrXIV	241142	-	16.16
NUT0699	chrXV	343227	+	16.15
SNR48	chrVII	609769	+	15.96
CUT905	chrXVI	622923	-	15.91
CUT340	chrXIV	568559	+	15.88
NUT0558	chrXIII	13774	+	15.85
NA	chrVII	559366	+	15.84
CUT823	chrXV	101061	-	15.84
CUT812	chrXIV	434412	-	15.70
NUT0741	chrXV	930774	+	15.68
NUT1077	chrVII	894104	-	15.64
NUT0896	chrIV	443965	-	15.62
CUT260	chrXII	416075	+	15.55
CUT571	chrV	544874	-	15.55
NUT0160	chrIV	1297331	+	15.53
SNR10	chrVII	346294	+	15.25
CUT214	chrX	458662	+	15.21
CUT614	chrVII	671740	-	15.20

CUT399	chrXVI	41047	+	15.15
TYE7	chrXV	978041	-	15.14
NA	chrVIII	385235	+	15.09
NUT0855	chrII	786570	-	15.07
NUT0616	chrXIII	784645	+	15.02
NUT0322	chrVIII	68055	+	14.90
CUT861	chrXV	781423	-	14.89
CUT064	chrIV	502101	+	14.87
NUT1387	chrXV	79594	-	14.85
SNR71	chrVIII	411471	+	14.81
SNR83	chrXIII	626668	+	14.74
KTR2	chrXI	557877	+	14.65
NUT0256	chrVII	320259	+	14.59
GLT1	chrIV	155619	-	14.57
SNR61	chrXII	794287	-	14.54
NUT0053	chrII	615689	+	14.46
CUT374	chrXV	422133	+	14.38
CUT178	chrVIII	198778	+	14.37
NUT1523	chrXVI	882679	-	14.36
HUT1	chrXVI	87231	-	14.32
CUT051	chrIV	163549	+	14.31
NUT1384	chrXV	39983	-	14.22
CUT921	chrXVI	878303	-	14.16
NUT0959	chrV	115713	-	14.01
NUT0841	chrII	391186	-	13.97
CUT066	chrIV	563451	+	13.91
NUT1081	chrVII	958783	-	13.79
CUT744	chrXII	672513	-	13.76
NUT0932	chrIV	1183201	-	13.65
CUT015	chrII	326655	+	13.65
NUT0570	chrXIII	242291	+	13.60
CUT684	chrX	267983	-	13.60
CUT585	chrVII	55979	-	13.38
NUT0243	chrVII	64053	+	13.22
NUT0249	chrVII	173558	+	13.09
ERT1	chrII	699851	-	12.95
SNR13	chrIV	1403164	+	12.79
NUT0361	chrIX	136596	+	12.77
SNR128	chrX	139538	-	12.55
CUT368	chrXV	316180	+	12.50
CUT354	chrXV	22266	+	12.40
OLA1	chrII	291733	-	12.29
NUT0463	chrXI	446937	+	12.24
CUT733	chrXII	265028	-	11.80
NUT0117	chrIV	506605	+	11.75
SNR3	chrX	664097	+	11.73
NUT0942	chrIV	1321655	-	11.50
NUT1325	chrXIII	807785	-	11.48
NUT0363	chrIX	202534	+	11.46

CUT468	chrIV	22206	-	11.42
SNR30	chrXII	199445	+	11.36
SNR69	chrXI	365002	+	11.35
SNR31	chrXV	841952	-	11.29
NUT0218	chrVI	16922	+	11.27
CUT418	chrXVI	432564	+	11.27
CUT680	chrX	172782	-	11.04
CUT644	chrVIII	231468	-	10.98
KAP95	chrXII	823355	-	10.87
SNR36	chrXV	680685	-	10.85
NUT0861	chrIII	124701	-	10.70
SNR42	chrXI	558935	-	10.54
NUT1114	chrVIII	455034	-	10.48
NA	chrXI	317694	+	10.25
NUT0763	chrXVI	95857	+	10.24
NUT0554	chrXII	1002486	+	10.11
SNR82	chrVII	317156	+	9.75
NUT0310	chrVII	1060500	+	9.63
SNR63	chrIV	323229	-	9.01
CUT775	chrXIII	513189	-	7.96
SNR5	chrXV	842699	+	7.81
CUT901	chrXVI	481060	-	4.09
